# Single Institution’s Dosimetry and IGRT Analysis of Prostate SBRT

**DOI:** 10.1186/1748-717X-8-215

**Published:** 2013-09-13

**Authors:** Q Jackie Wu, Taoran Li, Lulin Yuan, Fang-Fang Yin, W Robert Lee

**Affiliations:** 1 Department of Radiation Oncology, Duke University Medical Center, Box 3295, Durham, NC 27710, USA

**Keywords:** Stereotactic body radiation therapy, SBRT, Prostate, IGRT, Clinical trial

## Abstract

**Background and purpose:**

To report single institution’s IGRT and dosimetry analysis on the 37 Gy/5 fraction prostate SBRT clinical trial.

**Materials/methods:**

The IRB (Duke University Medical Center) approved clinical trial has treated 28 patients with stage T1-T2c prostate cancer with a regimen of 37 Gy in 5 fractions using IMRT and IGRT protocols since 2009. The clinical trial protocol requires CT/MRI imaging for the prostate delineation; a margin of 3 mm in posterior direction and 5 mm elsewhere for planning target volume (PTV); and strict dose constraints for primary organs-at-risks (OARs) including the bladder, the rectum, and the femoral heads. Rigid IGRT process is also an essential part of the protocol. Precise patient and prostate positioning and dynamic tracking of prostate motion are performed with electromagnetic localization device (Calypso) and on-board imaging (OBI) system. Initial patient and target alignment is performed based on fiducials with OBI imaging system and Calypso system. Prior to treatment, cone-beam CT (CBCT) is performed for soft tissue alignment verification. During treatment, per-beam corrections for target motion using translational couch movements is performed before irradiating each field, based on electromagnetic localization or on-board imaging localization. Dosimetric analysis on target coverage and OAR sparing is performed based on key DVH parameters corresponding to protocol guidance. IGRT analysis is focused on the average frequency and magnitude of corrections during treatment, and overall intra-fractional target drift. A margin value is derived using actual target motion data and the margin recipe from Van Herk et al., and is compared to the current one in practice. In addition, cumulative doses with and without per-beam IGRT corrections are compared to assess the benefit of online IGRT.

**Results:**

1. No deviation has been found in 10 of 14 dosimetric constraints, with minor deviations in the rest 4 constraints.

2. Online IGRT techniques including Calypso, OBI and CBCT supplement each other to create an effective and reliable system on tracking target and correcting intra-fractional motion.

3. On average ½ corrections have been performed per fraction, with magnitude of (0.22 ± 0.11) cm. Average target drift magnitude is (0.7 ± 1.3) mm in one direction during each fraction.

4. Benefit from per-beam correction in overall review is small: most differences from no correction are < 0.1 Gy for PTV D1cc/Dmean and < 1%/1.5 cc for OAR parameters. Up to 1.5 Gy reduction was seen in PTV D99% without online correction. Largest differences for OARs are −4.1 cc and +1.6 cc in the V50% for the bladder and the rectum, respectively. However, online IGRT helps to catch unexpected significant target motion.

5. Margin derived from actual target motion is 2.5 mm isotropic, consist with current practice.

**Conclusions:**

Clinical experience of the 37 Gy/5-fraction prostate SBRT from a single institution is reported. Dosimetric analysis demonstrated excellent target coverage and OAR sparing for our first 28 patients in this trial. Online IGRT techniques implemented are both effective and reliable. Per-beam correction in general provides a small benefit in dosimetry. Target motion measured by online localization devices confirms that current margin selection is adequate.

## Background

In the last decade investigators from across the globe have examined a number of hypo-fractionated regimens for prostate radiation treatment, with daily fraction sizes ranging from 2.5 to 7.25 Gy delivered in 5–28 fractions over 1–6 weeks [1]. During the same period, a number of studies have been published that suggest that the α/ß ratio of prostate cancer may be in the range of 1.2 – 3.0 Gy [2]. In addition, it has also been suggested that the α/β ratio for late rectal toxicity (primarily bleeding) is in the range of 4–5 Gy [3]. If these hypotheses are indeed true then treating prostate cancer with fewer, larger fraction (to a lower total dose) may result in an increase in the therapeutic ratio [1]. Therefore, a new treatment paradigm is warranted for clinical investigation, assume it will not only limit the volume of normal tissue irradiated by using tighter margins but will also greatly decrease the overall treatment time and may provide a biologic basis to decrease rectal toxicity. It is hypothesized that hypo-fractionated radiation therapy using continuous real-time evaluation of prostate motion may offer the ability to reach each of these goals by limiting radiation dose to surrounding normal tissues while optimizing treatment to the prostate and taking advantage of differences in prostate cancer and normal tissue responses to radiation therapy to improve upon the therapeutic ratio.

The five-fraction prostate stereotactic-body radiation therapy (SBRT) protocol is developed under this hypothesis. Previously, several five-fraction prostate SBRT regimens has been developed and evaluated by clinicians and researchers at other institutions, and treatment outcomes were promising [4]. The 1st experience of five-fraction regimen for prostates was reported by Virginia Mason Clinic in 2006 [5]. A total of 40 patients with low risk prostate cancer were treated with 6.7 Gy per fraction. Bolzicco et al. [6] reported on treating 45 low-and intermediate-risk prostate cancer patients with Cyberknife® SBRT at the regimen of 35 Gy in five fractions. Katz et al. from Winthrop University Hospital, NY [7] reported a study with 304 patients all treated with 5 fractions using Cyberknife®. Total doses are 35 Gy for the 1st 50 patients and 36.25 Gy for the rest. Freeman and King [8] reported five-year outcomes on 41 low-risk prostate cancer patients receiving SBRT with CyberKnife® with total dose of 35–36.25 Gy in five fractions. Alongi et al. [9] reported a phase II study on linac-based prostate SBRT with VMAT technique based on 40 patients. The prescription is 35 Gy in 5 fractions. In terms of treatment outcome, no > Grade 3 toxicity was reported in any of the previous studies. Patients were reported to tolerate the SBRT treatment well, with biochemical control rate between 93% to 100% depends on the length of the median follow-up.

In this report, we present our experience of the institutional prostate SBRT clinical trial, with a focus on target motion statistics and the IMRT/IGRT techniques we implemented, and their combined impact on dosimetry.

## Materials and method

### Patient data and treatment planning

From 2009 to 2012, 28 stage T1-2 prostate cancer patients were enrolled in the IRB approved institutional SBRT clinical trial. Patients receive 5 fractions of radiation with each fraction size of 7.4 Gy and the total dose of 37 Gy. The 5 treatments are scheduled to be delivered every other day. A minimum of 36 hours and a maximum of 96 hours should separate each treatment. No more than 3 fractions will be delivered per week. The total duration of treatment will be no shorter than 10 days and no longer than 18 days.

Patients are asked to have a full bladder during simulation and daily treatment. Patients are given instructions to drink 16–24 oz of water or other fluid 2–3 hours prior to treatment and to not urinate between this time and treatment as they are able. For rectum filling management, patients are instructed to take one tablespoon of Milk of Magnesia the night and one Fleet’s enema 2–3 hours before the simulation and each treatment.

Computed Tomography (CT) is the primary image set for treatment planning. The simulation is performed in the supine treatment position, with immobilization device (alpha-cradle) to minimize body motion and rotation. Axial cuts of 1.25 mm are acquired throughout the pelvis for the accuracy of marker identification and localization. Magnetic resonance imaging (MRI) is also acquired and fused with CT images to assist target delineation. MRI is acquired prior to Calypso transponder implantation to exclude artifacts interfering with soft tissue contouring. For fiducial markers, MRI is acquired in the same day of planning CT to minimize patient visit to the clinic.

CT and MRI image sets are registered for the delineation of target volumes and OARs. The definition of volumes is in accordance with the ICRU Report #50 and ICRT Report #62: Prescribing, Recording, and Reporting Photon Beam Therapy [10,11]. The gross tumor volume (GTV) for the purposes of this protocol is the prostate only, and is defined by the physician based on the planning CT and MR along with clinical information. The clinical target volume (CTV) is the same as the GTV. The planning target volume (PTV) is defined as the CTV plus a 3 mm posteriorly and 5 mm in all other dimensions.

IMRT planning is performed in Eclipse® Treatment Planning System (Varian Medical Systems, Palo Alto, CA). 7 or 9 co-planar beams are used to deliver dose distribution that is highly conformal to the PTV while maximally spare adjacent OARs. The plans are normalized so that the prescription isodose line covers at least 95% of the PTV. Detailed dosimetric constraints of the protocol are listed in Table 1.

**Table 1:** Key dosimetric constraints used in the IMRT planning of this protocol

**Target (PTV)**	D1cc ≤ 43.0 Gy
	Dmean > 37.0 Gy
**Bladder**	D1cc < 40.7 Gy
	V37 Gy < 2 cc
	V24 Gy < 40 cc
**Rectum**	D1cc ≤ 39.3 Gy
	V37 Gy < 2 cc
	V33 Gy < 25% volume
	V28 Gy < 40% volume
	V24 Gy < 50% volume
**Femoral Heads**	V20 Gy < 10 cc
	D1cc < 30 Gy

### Imaging-guidance and dynamic tracking techniques

This SBRT protocol requires stereotactic treatment with the use of a 3-D coordinate system defined by implanted electromagnetic transponders or implanted fiducial markers. The initial localization and alignment is based on the center of mass of the transponders/fiducial markers. Prior to treatment, kV CBCT is also acquired for soft-tissue alignment verification and deformation review. Significant rotations or soft tissue deformations will be corrected at initial localization stage, and intra-fractional rotations are ignored. After initial localization is performed, all effort is made to initiate the treatment delivery as quickly as possible. Continuous tracking and adjustment of target position during treatment is achieved by translational shifting the center of mass determined via electromagnetic transponders or fiducial markers, using remote couch movement. Specifically, per-beam couch corrections is made, if necessary, based on the actual target motion provided by dynamic tracking with either Calypso or per-beam kV orthogonal imaging with OBI. A correction action is performed if the target migrated more than 3 mm in any of three orthogonal coordinates.

To analyze the IGRT effectiveness, the motion correction magnitude and frequency of each treatment fraction are pooled and analyzed. The overall drifting magnitude during treatment is also captured and used to perform the margin evaluation based on the margin recipe described by Van Herk et al. [12].

Dosimetric benefit of dynamic tracking and online correction is evaluated by comparing delivered dose distributions with and without such correction. The dose distribution without correction is generated in the same treatment planning system by accumulating doses without online correction from each beam. The difference in dosimetry between actual treatment with online IGRT and simulated treatment without per-beam online corrections is used to quantify the benefit of using dynamic tracking and correction. The dosimetric analysis was based on original planning CT and contours.

## Results and discussions

### Treatment plan Dosimetry

The dosimetry of 28 treatment plans and their compliance to the protocol constraints are summarized in Table 2. The CTV is very well covered for all 28 patients. Majority of dosimetric parameters are well within protocol constraints (Table 1); no constraints are exceeded in PTV and femoral heads. For the rectum, 1 case has Dmax exceeding constraint value 39.3 Gy by 0.03 Gy; and 3 cases have V37 Gy exceeding constraint value 2 cc by 0.08, 0.60 and 0.02 cc, respectively. For the bladder, 1 case has V24 Gy exceeding constraint value 40 cc by 4.6 cc; and 18 cases have V37 Gy exceeding constraint 2 cc by (3.1 ± 3.1) cc.

**Table 2:** Key dosimetric parameter statistics

	**Structure volume**	**Key DVH parameters statistics**		**# of patients w/minor deviations**	**Deviation amount**
**CTV**	(53.6 ± 24.1) cc	Dmean	(102.4 ± 1.1) Gy	N/A	N/A
		Dmax	(104.8 ± 1.7) Gy		
		Dmin	(100.2 ± 1.4) Gy		
		D99%	(100.2 ± 1.0) Gy		
**PTV**	(98.8 ± 36.8) cc	D1cc	(39.1 ± 0.6) Gy	0	
		Dmean	(37.9 ± 0.3) Gy	0	
		D99%	(36.1 ± 0.5) Gy	0	
		CI (RTOG)	(0.99 ± 0.02)	0	
**Bladder**	(228 ±144) cc	Dmax	(38.6 ± 0.5) Gy	0	
		V37 Gy	(3.6 ± 3.0) cc	18	(3.1 ± 3.1) cc
		V24 Gy	(20.0 ± 10.8) cc	1	4.6 cc
**Rectum**	(69.1 ± 27.0) cc	Dmax	(38.3 ± 0.6) Gy	1	0.03 cc
		V37 Gy	(1.0 ± 0.7) cc	3	0.08, 0.02, 0.6 cc
		V33 Gy	(4.3 ± 2.1) cc	0	
		V28 Gy	(8.7 ± 3.6) cc	0	
		V24 Gy	(11.2 ± 4.7) cc	0	
**Femoral Heads**	-	D1cc	(17.8 ± 2.70) Gy	0	
		V20 Gy	(0.3 ± 1.3) cc	0	

### IGRT analysis and margin evaluation

**Figure 1:**
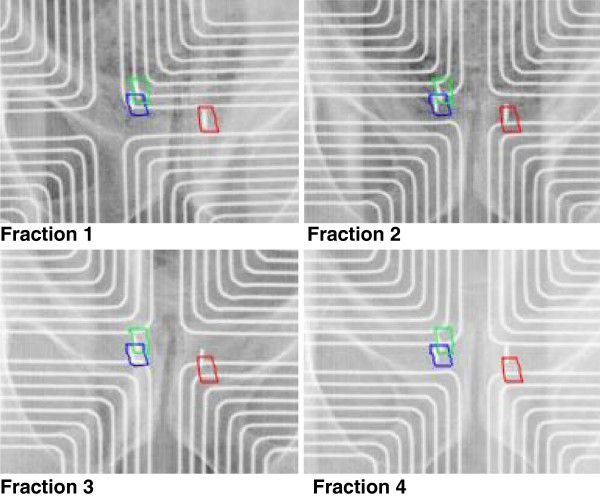
**Calypso transponder shifted position over the course of treatment.** The grid-like patterns in each image are the Calypso antenna, whose location relative to the patient is somewhat arbitrary for each fraction.

For the total of 28 patients, 25 were setup based on with Calypso and 3 were setup based on with OBI. Among the 25 Calypso cases, 4 patients had at least 1 or more fractions switched to OBI imaging due to technical issues. 5 (out of 28) patients had transponder/seed migration that was captured by Calypso/OBI or CBCT imaging and their corresponding locations were adjusted and monitored by CBCT. Figure 1 shows an example of such case. 3 (out of 28) patients (1 fraction each) had significant motion or soft tissue deformation that led to the treatment halt for that specific fraction. One had to wait till full bladder and empty rectum; one had muscle spasm that caused sudden large body motion and was able to maintain position after break; and one had unexplainable large body motion during treatment and was able to maintain position after communications and further instructions. Overall, the combination of Calypso, OBI and CBCT IGRT options not only supplements each other with their own unique advantages, but also helps to guarantee uninterrupted treatment when one option is not available.

**Figure 2:**
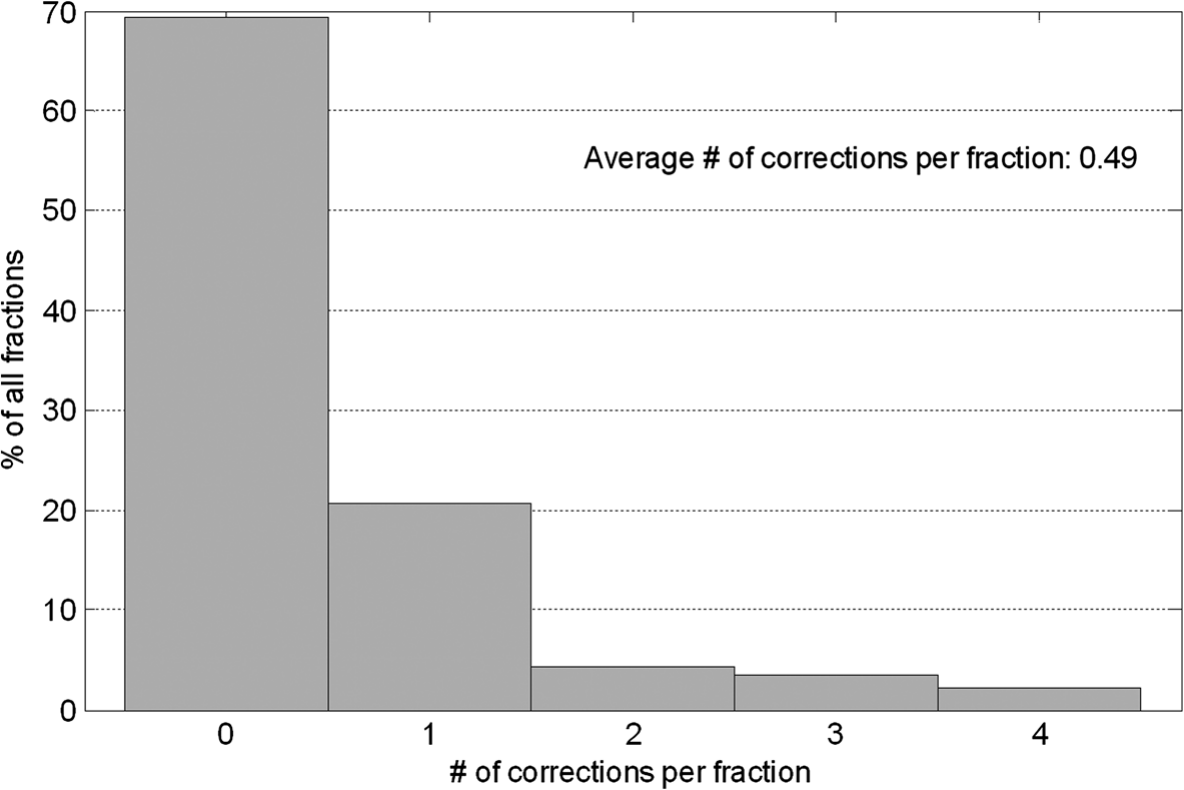
Histogram of per-fraction online correction frequency.

Online correction frequency per fraction is summarized in the histogram in Figure 2. Nearly 70% of fractions are treated without corrections; and ~90% fractions have less than 2 online corrections performed during treatment. On average 0.5 corrections have been performed per fraction.

**Figure 3:**
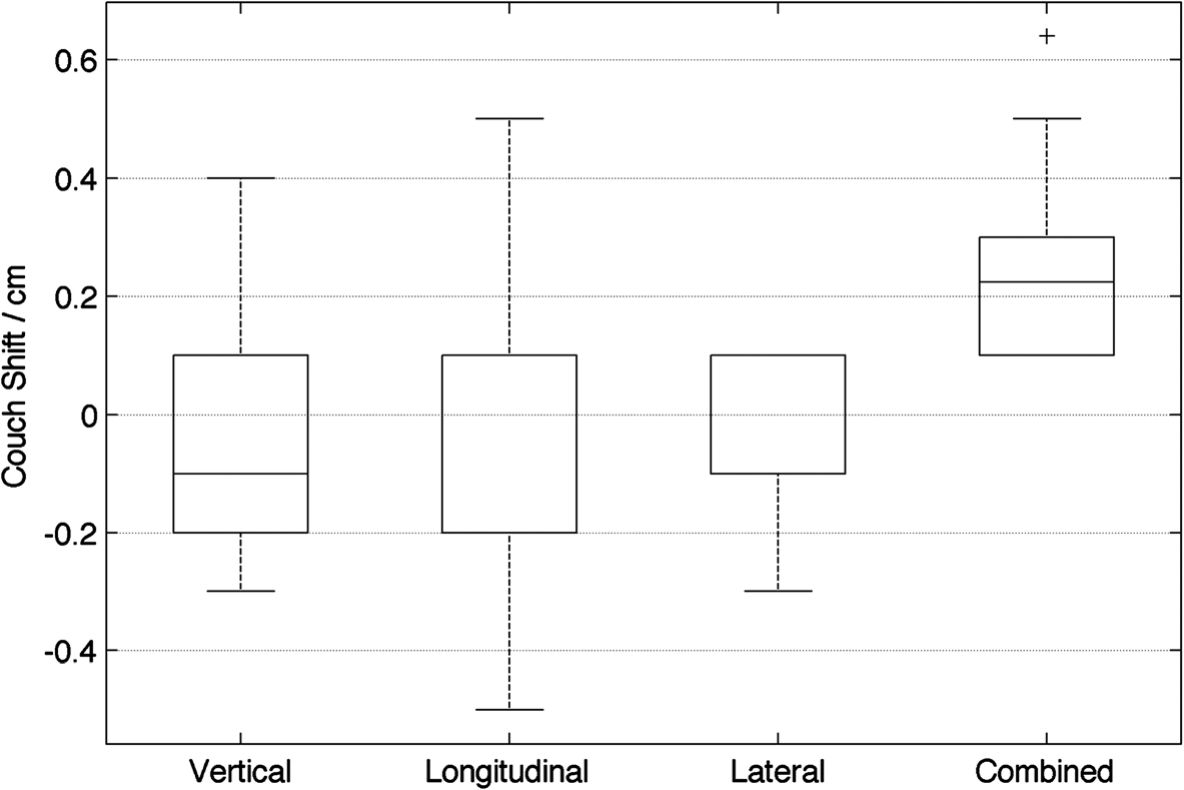
Boxplots of online correction magnitude in vertical, longitudinal and lateral directions, as well as combined length (added in quadrature).

The correction magnitudes in three directions (vertical, longitudinal and lateral) are shown in the first three box plots of the Figure 3, and the combined vector length of corrections are shown in the “Combined” boxplot. In general the corrections are small in all directions, with the lateral direction having the smallest correction range, and the longitudinal (superior-inferior) direction having the largest correction range. The combined correction magnitude from all three directions is (0.22 ± 0.11) cm.

**Figure 4:**
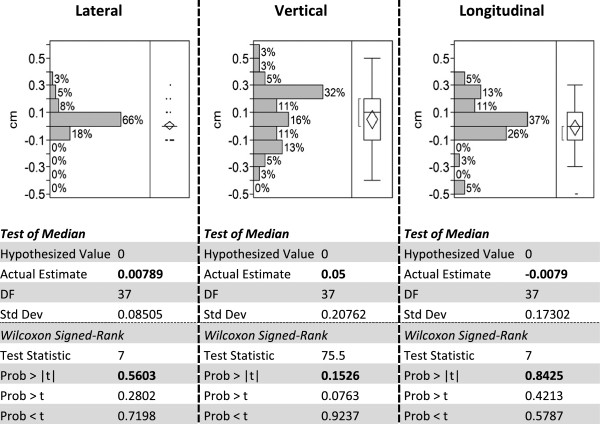
Histograms and Wincoxon Signed-Rank test results of target position drifts in Lateral, Vertical and Longitudinal directions.

The total couch shift from the start to the end of each treatment represents the target position drift. Histograms and statistical test results of target drift in three directions are shown in Figure 4. The magnitude is larger than corresponding values in Figure 3 because it shows the cumulative drift of the target during each fraction. Largest drifts are seen in couch vertical (AP/PA) direction, whereas lateral drifts are the smallest. Although occasional drifts > 0.3 cm can be seen, the Wincoxon Signed-Rank test reveals that drifts in none of the three directions are statistically significant.

In additional to the statistics above, the necessary margin for this specific patient group is calculated based on the target drift data described above and the margin recipe by Van Herk et al. [12]. The directional margins for AP/LR/SI directions are 2.5/1.0/1.7 mm; and the isotropic margin is 2.5 mm. This calculated margin size confirms that the protocol margin selection is sufficient.

### Dynamic tracking benefits analysis

**Figure 5:**
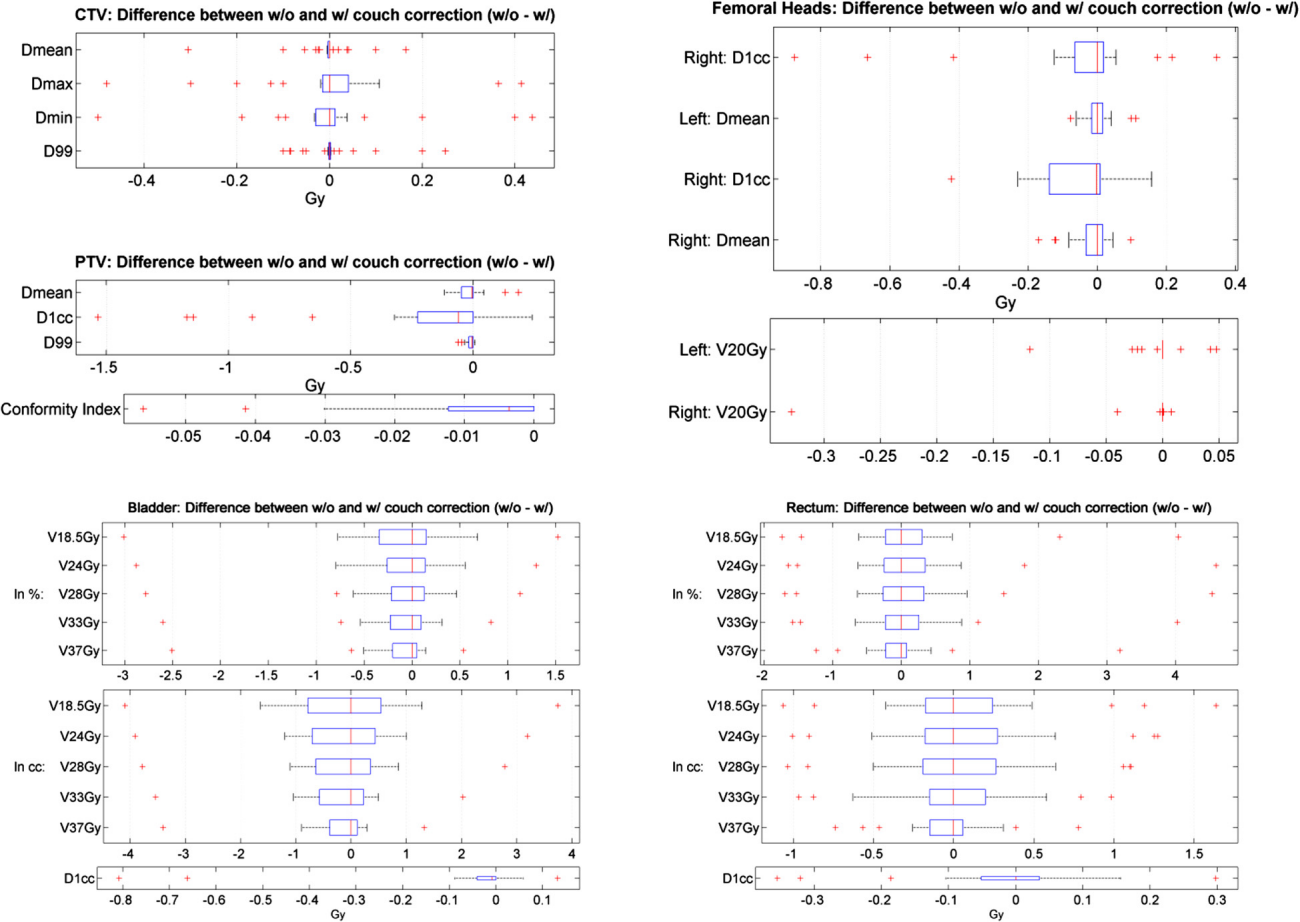
Differences in key dosimetric parameters between actual treatment with online IGRT and simulated treatment without per-beam correction.

Figure 5 shows the difference in multiple dosimetric parameters between the simulated treatment without online corrections and the actual treatment that includes the dynamic tracking and correction. In general, when looking at the cumulative dose over 5 fractions, the benefit from per-beam correction is small for all dosimetric parameters corresponding to the protocol constraints. The per-beam correction has very small impacts on CTV coverage: key CTV dosemetric parameters without corrections are all within ± 0.5 Gy (1.4%) from those calculated with per-beam corrections. Differences from no correction are < 0.2 Gy for PTV D1cc/Dmean and < 0.07 for both RTOG [13,14] and Paddick [15] conformity indices. Slightly higher difference is seen for PTV D99%: 0.6 to 1.5 Gy reductions are found for 5 patients without online correction. For the bladder, the majority of patients have < 1%/1.5 cc difference between with and without corrections, with only 2 patients having differences of −3%/-4.1 cc and +1.5%/+3.7 cc respectively. Similar distribution has been observed for the rectum: the majority has < 1%/< 0.7 cc difference, with exceptions of 4 patients having differences ranging from −1.7%/-1.1 cc to +4.6%/1.6 cc. Very small differences (< 0.9 Gy/< 0.4 cc) are also seen for the femoral heads dosimetric parameters. In general, only 1–2 patients (4-7%) would see >1%/1 cc increase in OARs irradiated volumes if no online correction was made. The rigorous supportive measures on bladder and rectum management that patients follow on each fraction and precise initial soft-tissue based target alignment have helped to minimize the motion during the radiation treatment. The variation could be larger and more frequent without these steps. The small diffrence between treating with and without online IGRT can be attributed to two other important components in the protocol. One is the carefully constrcucted immobolization device that helps to minimize the inter-fraction and the intra-fraction external patient body motion and rotation. Therefore, the online IGRT can be focused on tracking and correcting internal organ motion. With patient education and instruction, the internal motion as obseverd, can be kept to a small range and overall target drfiting is small too.

On the other hand, the dynamic tracking helps to catch the significant motion in those 3 patients/fractions that requires beam off/treatment halt. Currently, there is no clear indication on complete prevention of such sudden motion, thus the dynamic tracking is still beneficial to ensure the patient is treated with high precision through each beam irradiation.

In this study all dosimetric analysis was performed on the planning CT and original contours. In reality patient’s anatomy is dynamic and could change from day to day; therefore more accurate dosimetric analysis could be achieved if daily anatomical information is taken into consideration through online imaging modalities such as CBCT. However at current stage, due to the suboptimal image quality of CBCT images and lacking of a reliable deformable image registration tool, accurate cumulative dose analysis of the bladder and the rectum using daily anatomy is still difficult to achieve. We are actively working towards this goal though in-house algorithm development and close collaborations with vendors.

## Conclusion

This report summarizes our first 3 year experiences on a 37 Gy/5-fraction prostate SBRT clinical trial, with a focus on target motion statistics and the IMRT/IGRT techniques we implemented, as well as their combined impact on dosimetry. Dosimetric analysis demonstrated excellent target coverage and OAR sparing for our first 28 patients in this trial. The majority of DVH parameters are well within protocol constraints. Online dynamic tracking with Calypso and/or OBI is both reliable and effective, resulting in 0.49 target position corrections per treatment fraction. These per-beam corrections provide a small benefit in dosimetry, but help to retain target coverage when large target motion occurs. Target motion measured by online localization devices is generally small, and confirms that the current margin selection is adequate. Future protocol analysis will include GI/GU toxicity and disease-free survival when follow up time is sufficient.

## Competing interest

The authors declare that they have no competing interest.

## Authors’ contributions

WRL is the PI of the clinical trial and the attending physician who supervised treatments in the study, including approving the treatment plans and all soft-tissue matching during the IGRT process. QJW is the co-investigator and the physicist of the clinical trial, in charge of data collection, treatment planning and IGRT/online monitoring components. TL performed data collection, analysis and simulation of treatments without online corrections. LY and FFY both contributed to data collection and analysis. All authors contributed to drafting the manuscript. All authors read and approved the final manuscript.
